# Surgical Guidance for Removal of Cholesteatoma Using a Multispectral 3D-Endoscope †

**DOI:** 10.3390/s20185334

**Published:** 2020-09-17

**Authors:** Eric L. Wisotzky, Jean-Claude Rosenthal, Ulla Wege, Anna Hilsmann, Peter Eisert, Florian C. Uecker

**Affiliations:** 1 Department of Computer Vision and Graphics, Fraunhofer Heinrich-Hertz-Institute, 10587 Berlin, Germany; jean-claude.rosenthal@hhi.fraunhofer.de (J.-C.R.); ulla.wege@hhi.fraunhofer.de (U.W.); anna.hilsmann@hhi.fraunhofer.de (A.H.); peter.eisert@hhi.fraunhofer.de (P.E.); 2 Department of Visual Computing, Humboldt Universität zu Berlin, 10117 Berlin, Germany; 3 Department of Otorhinolaryngology, Charité-Universitätsmedizin Berlin, 10117 Berlin, Germany; fc.uecker@charite.de

**Keywords:** stereoscopic imaging, multispectral imaging, image-guided surgery, surgical guidance, clinical decision support

## Abstract

We develop a stereo-multispectral endoscopic prototype in which a filter-wheel is used for surgical guidance to remove cholesteatoma tissue in the middle ear. Cholesteatoma is a destructive proliferating tissue. The only treatment for this disease is surgery. Removal is a very demanding task, even for experienced surgeons. It is very difficult to distinguish between bone and cholesteatoma. In addition, it can even reoccur if not all tissue particles of the cholesteatoma are removed, which leads to undesirable follow-up operations. Therefore, we propose an image-based method that combines multispectral tissue classification and 3D reconstruction to identify all parts of the removed tissue and determine their metric dimensions intraoperatively. The designed multispectral filter-wheel 3D-endoscope prototype can switch between narrow-band spectral and broad-band white illumination, which is technically evaluated in terms of optical system properties. Further, it is tested and evaluated on three patients. The wavelengths 400 nm and 420 nm are identified as most suitable for the differentiation task. The stereoscopic image acquisition allows accurate 3D surface reconstruction of the enhanced image information. The first results are promising, as the cholesteatoma can be easily highlighted, correctly identified, and visualized as a true-to-scale 3D model showing the patient-specific anatomy.

## 1. Introduction

Cholesteatoma is a disease of the middle ear. It consists of sprawling squamous epithelium. It is not cancerous, but cholesteatoma can lead to life-threatening complications due to its destructive growth. Therefore, the growth needs to be treated by surgery, which is currently the only possible treatment. In addition, cholesteatoma requires a complete resection of affected tissue to avoid a recurrence. Especially, the proliferation of this epithelium in the middle ear cavity and further growth into the mastoid and lateral skull base can lead to life-threatening complications. The damage of adjacent structures, as the ossicular chain and the cochlea, can cause hearing loss and lead to deafness. Furthermore, facial paralysis might be caused by erosion of the facial canal and disturbances of the equilibrium organ due to detraction of the semicircular fistulas. Fortified inflammation causes mastoiditis, meningitis, intracranial abscesses, and sinus vein thrombosis [1,2,3,4,5,6,7,8].

Surgery as the only treatment has the objective of the complete removal of cholesteatoma while recovering or preserving the hearing ability. In order to avoid revision surgery due to non-removed/non-identified cholesteatoma tissue, the complete removal of cholesteatoma is superordinate to the other objectives, such as the preservation of hearing ability. In cases of residual and recurrent cholesteatoma, a revision surgery is strictly needed [9,10,11,12]. All existing techniques of extensive cholesteatoma surgery include a form of mastoidectomy. The removal of the external auditory canal is considered to be the most effective procedure to allow complete cholesteatoma removal. However, the preservation or restoration of the hearing ability at the same time requires a high level of surgical expertise [5,8,13,14,15].

Digitization creates new possibilities to support the surgeon in such complex surgical procedures. On the one hand, digital stereoscopic image acquisition allows three-dimensional (3D) reconstruction of the situs. Based on 3D reconstruction results, contactless and radiation-free metric measurements of the anatomical structures can be performed [16]. In addition, spectral imaging can be used to detect optical tissue properties that are normally indistinguishable to the human eye [17]. All this additional knowledge has the potential to support the surgeon in his/her decision making and facilitate the surgical procedure when appropriate intraoperative real-time visualizations are used. Thus, these new image-based methods have the potential to accelerate complex surgical procedures and avoid re-surgery for an improved patient outcome [18].

In contrast to computer tomography (CT) and magnetic resonance imaging (MRI) methods, radiation-free image-based 3D reconstruction enables a continuous creation of 3D models of the patient-specific anatomy. In general, this 3D data model the exterior surface information of the analyzed volume and can be visualized as a point cloud or as a mesh representation. 3D measurements in combination with the spectral documentation of the cholesteatoma dimensions allow a true-scale comparison to preoperative CT data [16,19,20].

Multispectral analysis is established in biomedicine for cell and skin analysis [21,22]. Important contributions for clinical and surgical applications using multispectral techniques were presented in [23,24]. However, multispectral analysis has not yet been used widely in intraoperative medical therapy for accurate image-guided tissue differentiation. Different techniques exist to acquire the spectral information of the present tissues. Hyperspectral line scanning or multispectral snapshot cameras can be used, where the spectral separation is achieved by filtering the light that reaches the imaging sensor [25,26]. To perform the spectral separation on the illumination side, e.g., filter-wheels can be used [27]. Since only a few specific wavelength bands are chosen for this study, the filter-wheel technique was selected.

The aim of this project is to build a multispectral 3D-endoscopic setup that allows the intraoperative analysis of cholesteatoma. The analysis results will be augmented to endoscopic image data with further information, easing the process of tissue differentiation and supporting the surgical decision-making process during the treatment. The possibility of sequentially switching between broad-band and narrow-band illumination opens up new possibilities with regard to intraoperative tissue-specific visualizations. In this study, the cholesteatoma in the middle ear of three patients are analyzed to sketch the feasibility of the outlined stereo-spectral imaging system.

## 2. Materials and Methods

### 2.1. Imaging Setup

**Figure 1:**
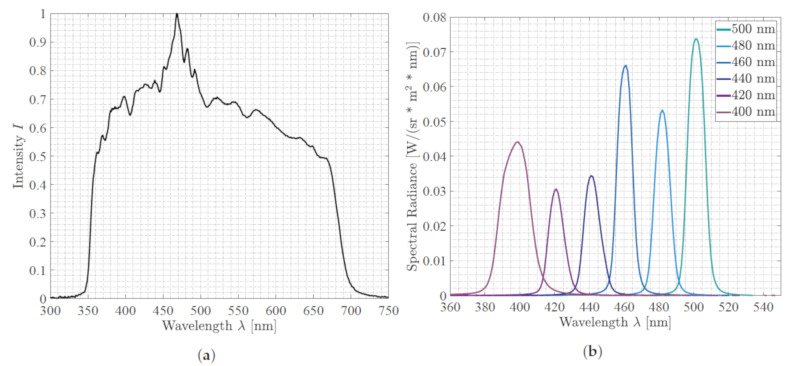
These plots show (**a**) the spectrum of the Xe illumination source, which is used for broad-band illumination of the situs, as well as (**b**) the six filters used from 400 nm to 500 nm for the narrow-band illumination mode. The Xe light source (**a**) contains cut-off filters at λ=350 nm, as well as λ=700 nm, and the illumination intensity *I* is normalized. In (**b**), the specific spectral radiance for each filter is presented to show the differences between the filter intensities.

**Figure 2:**
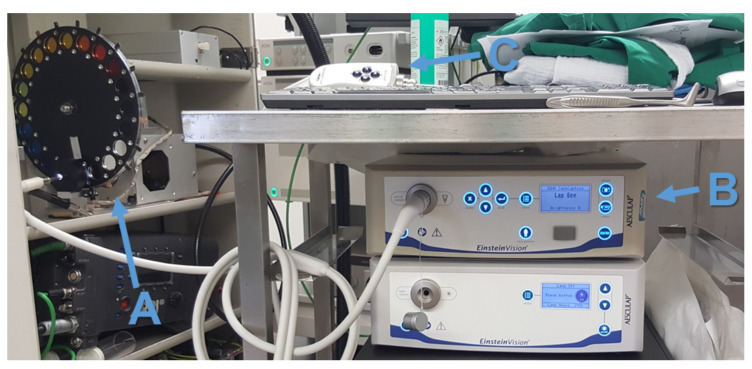
Stereo-spectral system setup: (A) the Xe light source with the filter-wheel, (B) the capturing unit, and (C) the endoscopic head.

We used a 3D laparoscope as our endoscopic imaging system (Schölly Fiberoptic GmbH, Denzlingen, Germany). The 3D-endoscope is a chip-in-scope system with an RGB Bayer pattern CMOS sensor. The sensor and camera specifications are listed in Table 1. The CMOS sensor used has a sensitivity range reaching from 380 to 1100 nm, and the focusing lens has an estimated focal length of 4.63 mm. The focus point is at 48 mm, which defines the optimal working distance (WD) to the surgical area. The external Xenon (Xe) light source holds a UV-filter to cut off the radiation below 350 nm, as well as an IR filter cutting the radiation above 700 nm; see Figure 1a. A filter-wheel is placed between the surgical scene and the Xe light source [28]. Thus, it becomes possible to select specific wavelength bands in the visible spectral range, from 400 nm to 500 nm in steps of 20 nm, by turning the filter-wheel; see Figure 2. The principle of the specific filter-wheel setup used in this study was described in Wisotzky et al. [17,29]. Each filter has a bandwidth in terms of a full-width half-maximum of about 20±3 nm. The light is guided through a fluid light-guide with optimal light transmission in the range of 340 nm to 800 nm, as the luminous efficiency is low due to the narrow-band filters, especially for 400 nm to 440 nm; cf. Figure 1b. The usage of a filter-wheel restricts the whole system, as real-time multispectral capturing is not possible and small camera movements can be present between the narrow-band images. We consider the current system setup as a proof-of-concept, as the aim of this work is to show the ability of differentiating cholesteatoma from surrounding tissue and combining this multispectral differentiation with volumetric situs data. A practical system would use a multi-spectral snap shot sensor with appropriate bands or synchronized lighting with different wavelengths.

During stereo-spectral image acquisition, it is essential to avoid indirect scattered broad-band illumination. Thus, other light sources need to be switched off during the acquisitions. However, due to the endoscopic design, the influence of scattered light during data acquisition can be minimized very effectively as the mastoid cavity is a very enclosed anatomical structure. The situs is alternately illuminated using the filter-wheel with the broad-band mode (light spectrum of Figure 1a) and narrow-band mode (light spectra of the six filters shown in Figure 1b). The surgical scene is captured with visible light (cf. Figure 1a) using the stereoscopic RGB sensor of the endoscope. This results in an image sequence of seven images of the same surgical view, one standard RGB image and six images holding only the reflectance of specific spectral illuminations in the range of 400–500 nm.

Furthermore, we extend the multispectral processing unit with a 3D reconstruction module to perform image-based measurements of the surgical scene. The combination of both methods enables us to compute a true-scale spectral 3D model of the patients’ anatomy, so that image-based volume and size measurements can be performed and registered to preoperative CT data. Nonetheless, a precondition to perform such image-based measurements and 3D reconstruction is a calibrated stereoscopic system. This includes the determination of lens distortion parameters, intrinsic parameters such as focal length and the principal point, as well as the estimation of the extrinsic parameters, i.e., the orientation of the two stereo cameras to each other and the inter-axial distance between both cameras. It is important to note that the working distance affects the accuracy of both techniques, 3D reconstruction and spectral tissue classification. Therefore, it is crucial to have an accurately calibrated endoscopic imaging system that compensates for distance dependent effects. Especially, stereo calibration is of high importance for photogrammetric applications and stereo image processing algorithms as they are highly coupled. In general, our camera calibration technique makes use of the so-called checkerboard pattern [30], which has been intensively discussed in the computer vision community for many applications including medical scenarios [31,32]. However, our calibration method differs from the well-known checkerboard methods in that we apply a model-based approach using image registration techniques to correlate captured patterns with the reference target plane [33].

Our 3D reconstruction pipeline consists of three steps: First, we apply a scene dependent rectification of image pairs via the detection of robust feature points [34]. Second, we perform a dense disparity estimation [35] using such rectified image pairs as the input. Disparity estimation fully works in parallel on a graphic card with CUDA support. The real-time procedure makes use of a statistical approach on the sub-pixel level to estimate new correspondences. These correspondences are distributed into the local neighborhood where new correspondences are determined within the next iteration. This independent propagation of new estimated correspondences guarantees that the whole image is constantly updated. The obtained dense disparity maps have sub-pixel accurate disparity values. Lastly, the corresponding points are 3D reconstructed from the sub-pixel positions using triangulation. Thus, we measure dimensions directly within the image to further support the decision-making process.

In Wisotzky et al. [29], a spectral calibration process was developed that uses monochromatic sensor data in combination with a filter-wheel setup. In this work, the approach is changed to RGB sensor data, where each color channel is used and calibrated independently. The analysis of the RGB data can be simplified as we only consider sensitive wavelengths within the spectral range of λ=400 nm to λ=500 nm. Therefore, we can omit the red and green channel during spectral illumination because no notable sensitivity is given. The red channel becomes sensitive at λ≈550 nm, and the green channel becomes sensitive at λ≥480 nm.

Thus, the main analysis is done only on the blue channel of the sensor, and all spectral bands are considered using the illumination λ within the defined spectral range. A similar approach was proposed in Wisotzky et al. [18] using a fully digital surgical microscope. In this study, the system is used to acquire all stereo-spectral information by rotating the filter-wheel. Then, the analysis of the data is done postoperatively. To avoid imaging errors induced by the filter-wheel, caused by movements or additional aberrational effects, the filter-wheel is stopped at each filter position, and the specific wavelength image is acquired [17,29]. This process does not allow real-time analysis, which is not the aim of this study, as we want to identify specific wavelengths usable for the application of cholesteatoma removal.

Despite the importance of the described calibration for robust and efficient surgical guidance, the optical lens properties of the system used are extremely relevant to understand captured image information and allow a detailed and correct analysis of the multispectral stereoscopic image data. This work investigates the introduced system in terms of its modulated transfer function (MTF) and different limitations of the optical system. The MTF is an industry standard to measure the imaging performance of the optical systems. Therefore, we use more than six spectral filters in the filter-wheel and extend the wavelength range up to λ=660 nm (eight additional filters) to get an improved understanding of the underlying optical properties of the whole system. Important limitations of an optical system, which are discussed in this study, are chromatic aberration and lens distortion. Both effects have strong influences on the measuring accuracy if the optical system is not calibrated in terms of spectral effects.

### 2.2. Surgical Cases and Tissue Specification

The patients analyzed in this study were a 58 year-old male and a 19 year-old and an 11 year-old female. The first two patients had a long history of recurrent cholesteatoma and consecutive surgical interventions (among others, canal wall down technique). The third patient presented cholesteatoma for the first time. All patients were introduced to us with a fetid runny ear, conductive hearing loss, and otalgia. Locally, both recurrent cholesteatoma patients had a large, moist radical cavity with insufficient drainage, and high facial spur was reduced in the two patients with the canal wall down technique.

**Figure 3:**
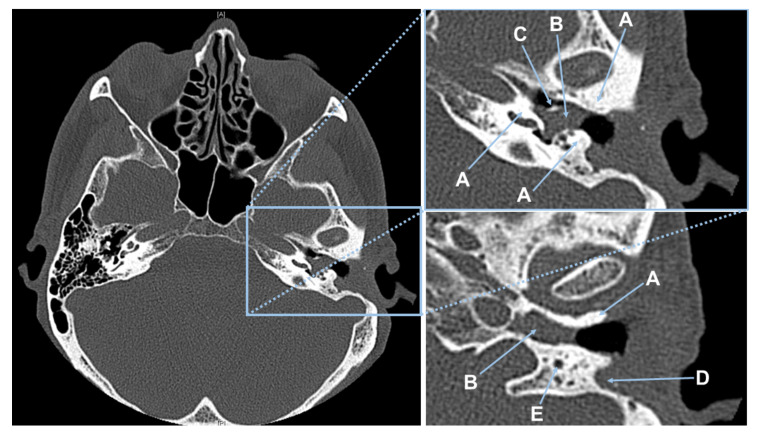
CT scan of Patient #1. (**Left**) Showing the complete CT slice. (**Top right**) Magnified CT-slice showing the patient’s left middle ear anatomy. In this view, (A) bone, (B) cholesteatoma, and (C) the handle of malleus covered with cholesteatoma. (**Bottom right**) Magnification of a 2nd CT-slice with (A) bone, (B) cholesteatoma, (D) connective tissue in the pre-surgical removed canal wall, and (E) the nervus facialis embedded in bone.

**Figure 4:**
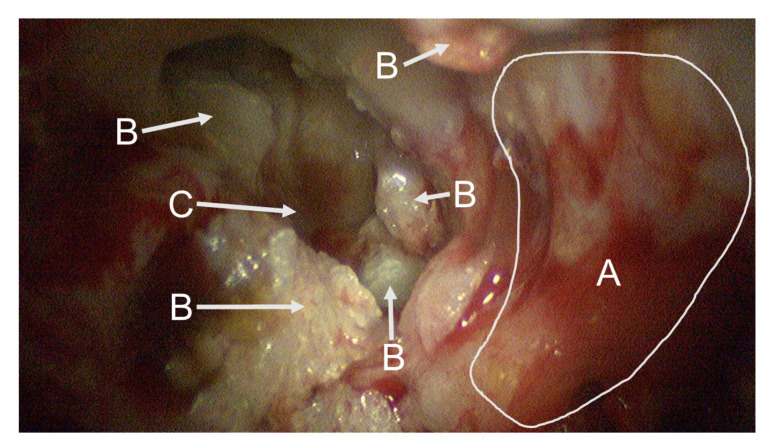
This endoscopic image shows the situs of the first patient captured at the beginning of the spectral data acquisition. In front, a large area of bone structure (A) is visible. Fragments of cholesteatoma (B) are present at different parts of the image. The round window niche (C) is visible in the background.

Intraoperatively, the cholesteatoma was confirmed microscopically with an expansion in the attic. Due to the migration theory and biologically destructive growth [36,37,38], as well as the history of multiple ear surgeries, the anatomy showed a broad osseous destruction with a missing ossicular chain and a partially removed ear canal wall including an infected mastoid cavity; see Figure 3. The recurrent cholesteatoma expanded probably from a residuum of the reconstructed tympanic membrane in a broad fashion over the round window niche, the intact stapes footplate in the attic and lateral to the facial nerve into the mastoid cavity; see Figure 4. Interestingly, the entire labyrinth block stayed inviolated. The cholesteatoma could be resected for all three patients and the high facial spur reduced. The radical cave was reconstructed by means of a concha cartilage graft, temporalis fascia, and a local flap plastic. The sound conduction could be rehabilitated by inserting a total ossicular replacement prosthesis (TORP) in the middle ear.

The written informed consent of acquiring stereo-spectral endoscopic data during the standard procedure was provided by all patients. The whole study was in agreement with the ethical approval obtained from the Ethics Committee of the Charité-Universitätsmedizin Berlin under Approval Code EA4/036/19.

### 2.3. Visualization

Multispectral imaging enables a measurement of spectral information for a complete scene if the camera has been accurately characterized and calibrated. The measured wavelengths λ are used to calculate spectral information for every captured surface area identifying interesting tissue types. In this specific case, the tissues-of-interest (TOI) are bone and cholesteatoma. Both tissue types appear white under normal broad-band illumination and RGB visualization, making a distinct tissue differentiation difficult.

Based on the knowledge from the spectrophotometer analysis of fresh tissue samples of the major present tissues (cholesteatoma and bone) [39], we can identify the best wavelength bands to amplify and highlight cholesteatoma tissue during the surgical treatment. It has been reported that the main difference of these two tissue types in terms of optical properties lies within the spectral range of λ=400 nm to λ=575 nm. In that spectral range, cholesteatoma shows a much higher reflectance than bone. Theoretically, cholesteatoma would appear much brighter compared to bone under illumination with λ=400 nm to λ=575 nm. According to that analysis and to avoid the spectral influences of blood, having peaks in the range of λ=555 nm [40], the described six appropriate spectral bands between λ=400 nm to λ=500 nm were selected. As further tissue types, e.g., connective tissues, can be present in the surgical area, the spectral behavior of these tissues has to be considered as well. According to different studies [41,42,43], other possible present tissue types show lower reflectance in the interval of λ=400 nm to λ=575 nm compared to cholesteatoma. In addition, these other tissue types do not appear white under normal light conditions and are easier to differentiate. Consequently, we want to visualize the additional relevant tissue information of the spectral range of λ=400 nm to λ=575 nm directly into the endoscopic image as an augmented overlay. Therefore, we propose the following basic concept for spatial color enhancement to highlight the identified cholesteatoma regions and maintain the original color balance and dynamic range. First, we remove endoscopic noise, which influences the spectral image quality for chosen wavelengths λ. We achieve this by transforming the spectral image into the frequency domain by applying a Fourier transformation. In the frequency domain, we do a simple low-pass filter to remove high frequency noise patterns. Second, we preserve the dynamic range, as well as the color balance in the reconstructed spectral RGB image by adding identified spectral tissue information to one channel only. As surgical images hold most of its information in the R (red) and G (green) channels, the additional spectral information are added to the B (blue) channel. This suits the circumstance that only the calibrated B channel information of the sensor response is used for the spectral analysis, as described in Section 2.1. After calibration, the intended straight-forward augmented overlay is achieved using:

(1) $$\left(\begin{array}{c} R^{res}\\ G^{res}\\ B^{res} \end{array}\right)=\left(\begin{array}{c} R^{bb}/\max\left(R^{bb}\right)\\ G^{bb}/\max\left(G^{bb}\right)\\ \left(B^{bb}/\max\left(B^{bb}\right)\right)+B_{cal}^{nb} \end{array}\right),$$

where $R^{bb},G^{bb},B^{bb}$ are the RGB sensor responses using broad-band (bb) illumination and $B_{cal}^{nb}$ represents the calibrated and modified B channel holding the relevant tissue information using narrow-band (nb) illumination:

(2) $$B_{cal}^{nb}\left(x\right)=\left\{\begin{array}{cc} y=\frac{B_{\lambda }^{nb}}{B_{\lambda }^{cor}} & \mathrm{if}\;y\geq t\\ 0 & \mathrm{if}\;y<t \end{array}\right.$$

with $B_{\lambda }^{cor}$ the correction image for the specific λ illumination and *t* a scene and image-specific threshold suppressing small reflectance responses based on the intensity distribution.

For intraoperative visualization, it is important to select only specific information that will be useful for the surgeon. In this work, we analyze the six spectral images to find the best wavelength band to differentiate between cholesteatoma and bone using the above augmented overlay strategy. Further, specular reflections can occur through surgical instruments and fluid accumulation (e.g., blood) on tissue areas and lead to unwanted sensor saturation. As specular reflections occur in every spectral image, these areas may be enhanced as well and appear bluish. For the surgeon, this may not be a problem, as instruments and fluid accumulations are easy to identify, but specular reflections can be detected and neglected during the visualization step. Specular image surfaces show a very low color saturation *S* and a very high color intensity *I*, while they are hue independent. Therefore, pixels can be easily classified as specular reflectance pixels using an effective threshold method:

(3) $$\begin{array}{cc} I & =\frac{R+G+B}{3}\\ S & =\left\{\begin{array}{cc} \frac{3}{2}\left(R-I\right) & \mathrm{if}\;(B+R)\geq 2G\\ \frac{3}{2}\left(I-B\right) & \mathrm{if}\;(B+R)<2G, \end{array}\right. \end{array}$$

where a pixel has to exceed the threshold of intensity I=0.8 and fall below the threshold of saturation S=0.1. Identified specular reflectance pixels are ignored during the visualization process.

## 3. Results

**Figure 5:**
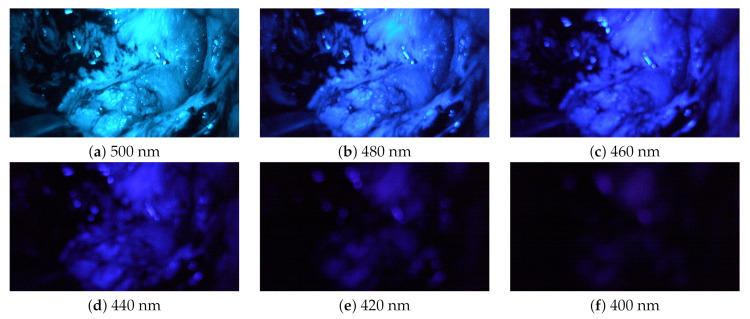
These images (**a**–**f**) show one complete captured sequence of the spectral images of Patient 2.

For each patient, two stereo-multispectral endoscopic videos are recorded showing the lateral skull base during the cholesteatoma removal procedure. The scanned situs of Patient 1 is shown in Figure 4. All captured data contain several filter-wheel sequences resulting in a set of twelve sequences in total. Figure 5 shows a complete sequence of six spectral images between 400 and 500 nm. As the sensor sensitivity is relatively small in the range of λ=380 nm to λ≈420 nm, the images with the filters of λ=400 nm and λ=420 nm hold less intensity than all other images, resulting in a fairly dark image, especially for λ=400 nm, and a very small signal-to-noise ratio (SNR).

### 3.1. Optical Properties of the Endoscopic System

The working distance of our stereo-multispectral system is adjusted on the normal broad-band illuminated RGB image to focus on anatomical structures with the highest interest; cf. Figure 4. In the spectral sequence, distortions and aberrations occur, especially for deep blue wavelength illumination in the range of λ=400 nm to 440 nm. The most prominent effect of this results in strongly blurred blueish spectral images. The blurring effect in these spectral images has two reasons: (1) undesired endoscopic camera movements and/or (2) various optical imaging errors. Undesired small movements may be present in the image as the surgeon is holding the endoscopic camera head, which can cause irregular small camera shakes and lead to misaligned spectral images in the sequence. Further, spectral images acquired with a filter-wheel setup are affected by different wavelength dependent imaging errors like chromatic aberrations, lens distortion, and light dispersion. In this work, all optical properties of the system, as well as all optical imaging errors are analyzed and discussed independently of each other. In this study, imaging errors are not induced by the movement of the filter wheel, because the filter wheel was stopped at each spectral measurement and remained in a static position. However, all effects influence each other and should be corrected in one λ dependent calibration step to achieve optimal image quality, but this process is beyond the scope of this paper.

#### 3.1.1. Modulated Transfer Function

**Figure 6:**
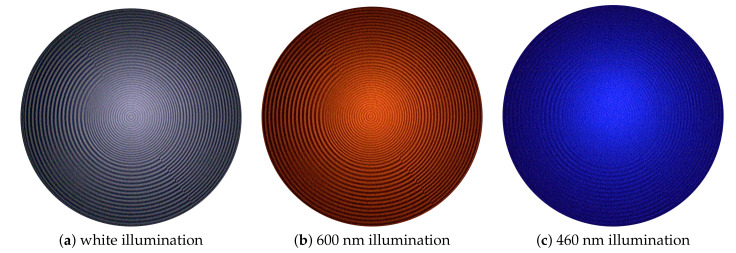
These images show multispectral filter-wheel acquisitions scanning the central circle pattern of the sharpness indicator by Putora [44]. (**a**) White light illumination and (**b**) 600 nm illumination: all circles are clearly visible, indicating a high degree of sharpness (DS) of 108.9 lp/mm; (**c**) 460 nm illumination: only thickest lines are visible with a computed DS of 62.3 lp/mm.

The degrees of sharpness (DSs) were analyzed using the type 7A9 sharpness indicator by Putora [44]. As shown in Figure 6, all circles are clearly visible for normal white light illumination (a), as well as illumination with λ=600 nm (b), indicating a DS of 108.9 lp/mm, while illuminating the pattern using the blue spectrum, e.g., with λ=460 nm (c), only gives 62.3 lp/mm. Medical images show mostly reddish image content; thus, the endoscopic optical system is optimized for λ in the range of around λ≈600 nm. For shorter blueish wavelengths, the lens design gets more complicated regardless of how narrow the spectral band used is due to glass materials tending to not perform well at shorter wavelengths [45].

The DS analysis is a first valuable indicator of the λ-dependence of the MTF. The MTF was analyzed using the Koren 2003 line test chart [46] and shows different behaviors for the imaging system using different wavelengths λ. The average 50% and 10% MTF frequencies for the blue spectrum of 400 nm to 460 nm are 38.75 lp/mm and 61.82 lp/mm, respectively, while for the range of 480 nm to 660 nm, the average 50% and 10% MTF frequencies are 49.0 lp/mm and 98.7 lp/mm. The 50% and 10% MTF frequencies of each specific filter can be found in Table 2. These results indicate that the images using blue illumination are not suitable to be used solely for visualization, but can be used as a source of specific spectral information only. Further, the reduced image quality makes image correction for the blue channels a major challenge.

#### 3.1.2. Chromatic Aberration

The 3D-endoscope has a fixed focus. The λ dependent refraction index of a lens causes chromatic aberrations. It includes transversal components [47,48], i.e., along the image plane, as well as longitudinal components [49,50], i.e., along the optical axis. This causes a chromatic focus shift for different wavelengths λ in relation to different working distances, i.e., the distance to the lens. Both aberrations, transversal and longitudinal, result in blurred spectral channels, which can be compared to a convolution applying a low-pass filter. The displacement of the focus plane is larger for small λ (e.g., λ=400 nm) than for greater λ (e.g., λ=500 nm), which corresponds to the image impressions of Figure 5 where the first two images (a) and (b) appear sharp, while the images (d) to (f), λ=440 nm to λ=400 nm, are blurry. This is caused by the lenses, which are optimized for sharp images between λ≈480 nm and λ≈650 nm. Accordingly, the focal shift increases in adjacent spectral regions.

#### 3.1.3. Lens Distortion

**Figure 7:**
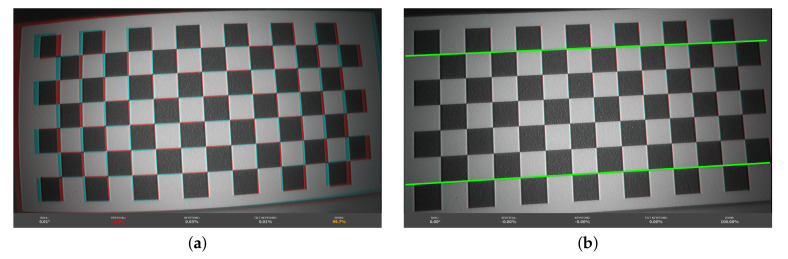
Stereo image pairs in anaglyph mode near the convergence plane showing error values before and after applying stereo lens distortion correction. (**a**) Distorted and non-rectified stereo image pairs. (**b**) Rectified stereo image pairs with eliminated lens distortion and correct visualization of straight lines.

Endoscopic imaging systems have a complex lens design due to the limited space and safety considerations in highly constrained environments. Therefore, endoscopes often come with wave distortions. This is a mixture of both simple distortion types, barrel and pincushion distortion, and can be caused by system designs to find a trade-off by trying to minimize distortion and maximize the field of view. This type of distortion shows a smaller deviation from the rectilinear projection, but is λ dependent [51]. The distortion increases with smaller λ. A calibration that removes that distortion requires special consideration of the different wavelengths. In our setup, the lens and stereo calibration is performed in the broad-band mode, and λ dependency is neglected for distortion correction, as depicted in Figure 7. Such distortion correction can be performed for each individual spectral channel, but the low image quality of the blue λ channels (cf. Section 3.1.1) does not allow this properly. Nonetheless, this shows the need to introduce a combined multispectral stereo/lens calibration to increase accuracy by incorporating the λ dependency.

#### 3.1.4. Penetration Depth of Light

The penetration depth of light rays is also λ dependent [52]. The higher λ is, the deeper the penetration into the tissue, which leads to higher scattering and depth aberration. Nonetheless, even depth aberration is λ dependent [53]. Depth aberrations arise from the refractive index mismatch between different tissue layers and types. They increase with decreasing λ. This might reduce the spatial image resolution and cannot be described and compensated analytically, as it depends on the geometry of the tissues and surfaces.

### 3.2. Cholesteatoma Visualization

All six images in Figure 5 show a similar behavior for cholesteatoma and bone. As expected, cholesteatoma is much brighter compared to bone. The images with λ=420 nm and λ=400 nm show the lowest intensity levels. The areas with captured intensity above the noise level correspond to identified cholesteatoma tissue. Due to this fact, we select λ=400 nm and λ=420 nm for further augmented visualization. However, it is also possible to use the narrow-band illumination options with the other wavelengths λ. Nonetheless, it is necessary to separate the regions of cholesteatoma (with higher intensity) and regions of bones (with lower intensity) with suitable computer vision methods, e.g., thresholding as one of the simplest methods. Further, specular reflections are present for higher λ, which makes a robust correction of this effect important.

**Figure 8:**
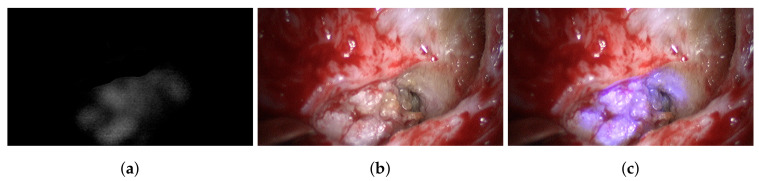
Captured data of Patient #2: (**a**) spectrally segmented cholesteatoma information with λ=420 nm illumination; (**b**) normal RGB view with broad-band illumination; (**c**) enhanced and augmented spectral RGB view.

**Figure 9:**
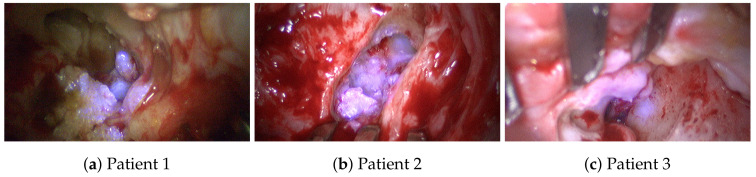
Augmented cholesteatoma visualization for the study group of three patients: (**a**,**b**) Visualizations using the spectral information at λ=420 nm. (**c**) Visualizations using the spectral information at λ=400 nm.

The B channel of the captured blue filter image is corrected using dark and white image information and then normalized. A resulting reflectance image is shown in Figure 8a. This image only holds information corresponding to cholesteatoma. This information is added to the B channel of the next recorded broad-band RGB image (Figure 8b) using Equation (1). The resulting image is shown in Figure 8c. In this enhanced view, it is easy to differentiate between different structures that have been white under normal broad-band illumination as some structures become bluish and others remain white. The bluish structures correspond to cholesteatoma, while the white structures are bone. This behavior is consistent over all analyzed data; see Figure 9. Thus, this enhanced view allows good surgical guidance to differentiate easily between these two tissue types while maintaining the decision process of the surgeon.

All marked regions identified by the surgeon as cholesteatoma in the broad-band images (cf. Figure 4) before and after spectral acquisition correspond to bluish structures in the improved RGB visualization. To confirm the diagnosis of the surgeon, all identified and removed tissue samples are analyzed using normal histological examination.

**Figure 10:**
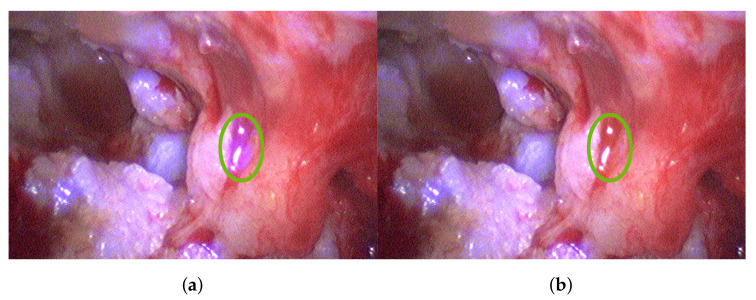
Importance of specular reflectance correction: (**a**) Enhanced RGB cholesteatoma visualization without specular correction showing wrongly detected regions. (**b**) Enhanced RGB cholesteatoma visualization with specular reflectance correction.

Specular reflectance can occur through the parts of the retractor, as well as fluid accumulation, reaching sensor saturation and leading to clipping. Therefore, local saturation correction can be necessary and omit over-saturated sensor areas. Figure 10a depicts such over-saturated regions. Accordingly, Figure 10b shows the clear benefit in adjusting saturation locally by omitting misleading information.

### 3.3. Multispectral Stereo Acquisition

**Figure 11:**
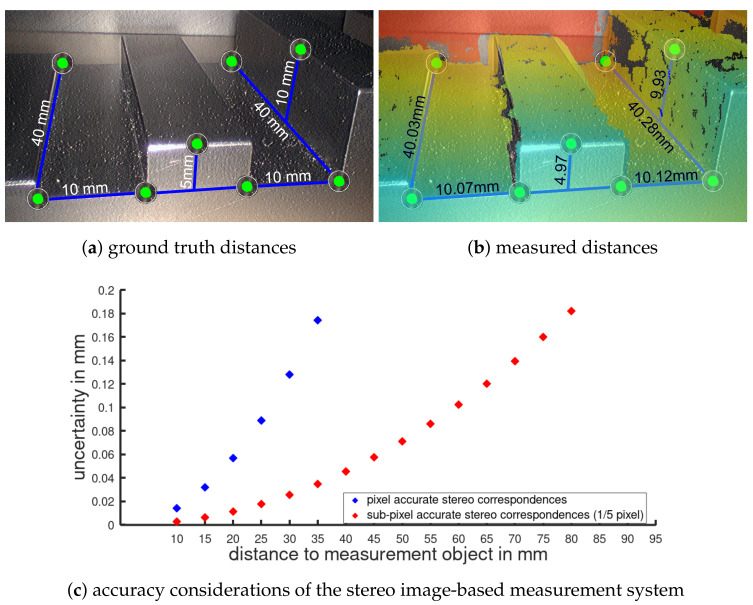
The comparison of the measured distances with ground truth data shows a high measurement accuracy: (**a**) specimen evaluation showing ground truth distances and (**b**) image based measurement results with color encoded depth information (red indicates far range and blue close range). The accuracy consideration in (**c**) shows an increased accuracy for larger working distances through sub-pixel (1/5) accurate stereo correspondences, which is of high importance for surgical intervention as typical working distances are between 20 and 60 mm.

**Figure 12:**
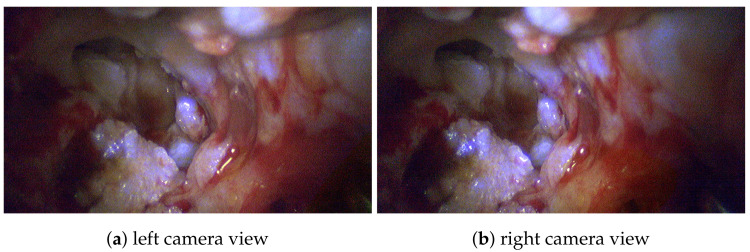
Stereo-spectral image pair with highlighted cholesteatoma tissue.

Besides the single advantages and benefits of stereoscopic and multispectral imaging, the combination of both results in a new valuable visualization of intraoperative data. The system design allows the 3D model generation of the patients’ anatomy, highlighting and identifying malicious tissue regions, and comparison to preoperative CT data. For such intraoperative volume analysis, the 3D reconstruction of the exterior surface measurements requires high accuracy. Evaluations using specimens with known dimensions give empirical accuracies of approximately 1/10 mm for several hundred point-to-point measurements; see Figure 11. Further, it has been shown in the context of visceral surgery that the image-based 3D reconstruction method is real-time capable and has one of the lowest error rates compared to similar approaches [54]. Figure 12 shows the stereoscopic input highlighting identified cholesteatoma for the left and right view, respectively. For both views, the relevant tissue could be consistently identified.

**Figure 13:**
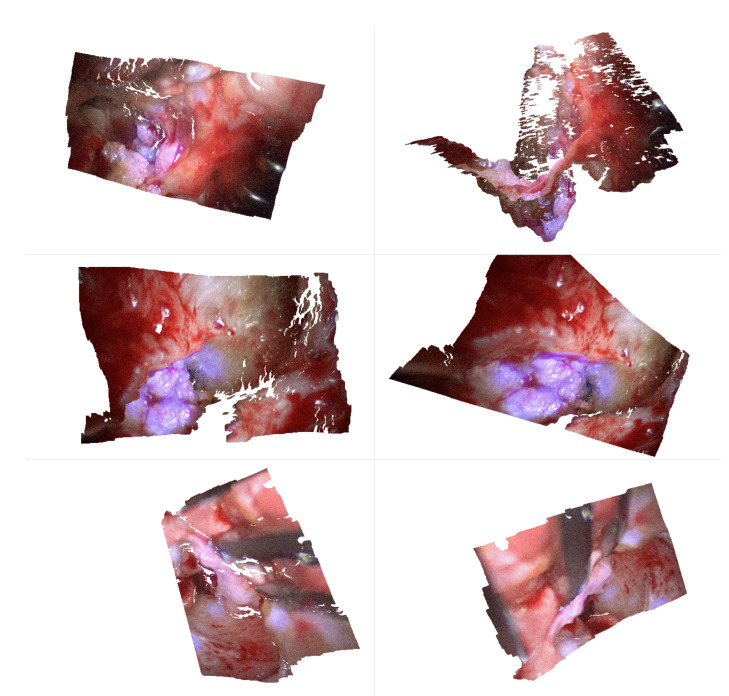
3D reconstructed spectral point cloud indicating spatial and depth related spread of cholesteatoma tissue. The point clouds consist of approximately 1.8 million vertices. Gaps in the 3D model occur from occlusion and missing stereo correspondences. (**Top row**) Patient #1; (**Middle row**) Patient #2; **Bottom row**) Patient #3.

**Figure 14:**
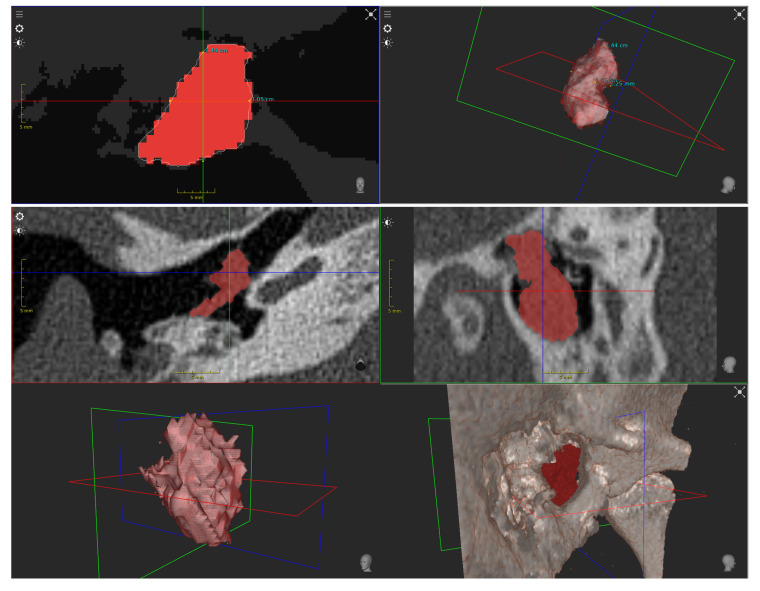
The preoperative CT segmentation from Patient #1 (**Top row**) and Patient #2 (**Middle and Bottom rows**) give a first good indicator about the size, shape, and volume of the cholesteatoma.

Then, we use the stereo-spectral image pair in Figure 12 for spectral 3D reconstruction to obtain a dense spectral 3D point cloud. The generated point clouds are very dense with a high level of anatomical detail and consist of approximately 1.8 million vertices. Figure 13 shows dense point clouds with different viewing angles for each patient of our study group. We can use this information for two interesting surgical diagnosis/treatment patterns, as the point cloud now contains the combined spectral and spatial information of the patient. First, we can highlight cholesteatoma areas in 3D and mask out non-relevant tissue areas, allowing a better understanding of the patients’ cholesteatoma size and shape. Second, tissue can be tracked and monitored in terms of how much volume has been removed from the middle ear anatomy during a procedure, e.g., the ear canal wall. For further surgical assessment, related preoperative CT segmentation results (Figure 14) can then be used in comparison to intraoperative 3D measurements.

## 4. Discussion

Besides the investigation of the spectral and physical sensor characteristic, we presented first options for surgical visualization adding multispectral information into RGB images without concealing relevant anatomical structures and features. In addition, the overall image and color impression is preserved, which allows the surgeon to intuitively understand the visually annotated surgical image showing the multispectral information. Moreover, strong local specular reflectance can be corrected to discard misleading information. However, this work presents a proof-of-concept for the surveillance of cholesteatoma removal with a focus on system analysis and selection of optimal wavelengths and less on perfect correction of spectral reflections.

As shown in the analyses of the optical properties, the image quality is strongly λ dependent. A focus adaption in combination with a λ dependent stereo calibration would allow a joint consideration of all lambda dependent and independent effects like distortion or aberration. This would then result in sharp images for all spectral illumination options and channels. Furthermore, we used the filter-wheel in this study only for analysis purposes, as it has some disadvantages, such as the lack of real-time capability [17]. Therefore, we suggest an adapted setup using a synchronized light source with white light and different blue flashes for clinical usage. Equally, the usage of a continuously running filter-wheel containing only the identified λ=400 nm and λ=420 nm, as well as empty spots for broad-band illumination seems feasible. In both cases, i.e., blue flashes and the filter-wheel, the blue images will not be present in the video stream independently and have to be processed to augment an overlay, as proposed in this work.

Multispectral tissue differentiation has become intensively studied and analyzed using machine learning methods as these can catch high-dimensional tissue behavior [55,56,57]. In this study, knowledge about the spectral behavior of cholesteatoma and bone, as well as only a few spectral bands are used. Therefore, differentiation using machine leaning seems not necessary, as this would need a large number of training data and would be computationally intensive compared to the usage of the discussed blue flashes.

Commercially, Karl Storz and Diaspective Vision have introduced an integrated multispectral endoscope [58]. Nonetheless, the system is still under development. Beside this new interesting approach, a common way to visualize specific anatomical structures is fluoroscopy, which depends on the insertion of indocyanine green (ICG) into the blood as a bio marker. Another commercially available method is narrow-band imaging, where differences between the blood oxygenation can be visualized [59]. Both methods can only highlight areas with sufficient blood flow. An enriched visualization of customizable structures (e.g. bone, fat tissue) is not possible with that approach, and therefore, a comparison seems difficult. Our presented method has the potential to visualize important tissue structures without introducing chemical agents into the patient. However, it seems feasible that commercially systems can achieve similar effects if it is possible to select similar wavelength bands and visualization options.

The combination of spectral visualization and 3D reconstruction opens up new possibilities for the integration of intraoperative and preoperative data, e.g., the comparison of the size, the shape, or removed tissue volumes. In conclusion, this work shows very promising results by means of stereo-spectral endoscopic-based tissue analysis using a sophisticated filter-wheel setup. It seems feasible that such a multimodal system can be integrated into a constrained environment like the operation room and open up new opportunities for intraoperative surgical assistance and image-guided interventions.

## Figures and Tables

**Table 1:** Specifications of the 3D-endoscope camera: sensor and calibrated lens values.

**Output Resolution**	Interlaced	1920 × 1080 px
**Frame Rate**		25 fps
**Endoscope Diameter**		approximately 9.4 mm
**Light Source**	Xenon (Xe)	300 W
**Focal Length**		4.63 mm
**Interaxial Distance**		4.16 mm

**Table 2:** The 50% and 10% MTF frequencies of all filters used.

Illumination	MTF 50%	MTF 10%
white	84.1 lp/mm	99.5 lp/mm
400 nm	40.2 lp/mm	65.8 lp/mm
420 nm	34.7 lp/mm	60.7 lp/mm
440 nm	35.9 lp/mm	57.8 lp/mm
460 nm	44.3 lp/mm	63.0 lp/mm
480 nm	51.0 lp/mm	98.4 lp/mm
500 nm	53.8 lp/mm	106.9 lp/mm
520 nm	47.9 lp/mm	98.4 lp/mm
540 nm	46.0 lp/mm	98.4 lp/mm
560 nm	47.4 lp/mm	98.5 lp/mm
580 nm	47.8 lp/mm	99.1 lp/mm
600 nm	52.0 lp/mm	98.7 lp/mm
620 nm	51.6 lp/mm	99.3 lp/mm
640 nm	47.0 lp/mm	94.9 lp/mm
660 nm	45.5 lp/mm	94.4 lp/mm

## Abbreviations

The following abbreviations are used in this manuscript:

RGB	Red-green-blue color model
CT	Computed tomography
MTF	Modulated transfer function
SNR	Signal-to-noise ratio
TOI	Tissue-of-interest
UV	Ultra violet
IR	Infrared
WD	Working distance
Xe	Xenon lamp
CMOS	Complementary metal oxide semiconductor
TORP	Total ossicular replacement prosthesis
